# Editorial: Fetal testicular hormones

**DOI:** 10.3389/fendo.2022.1090088

**Published:** 2022-11-21

**Authors:** Nathalie Josso, Rodolfo A. Rey, Andrew Pask

**Affiliations:** ^1^ Lipodystrophies, Adaptations Métaboliques et Hormonales, et Vieillissement, Sorbonne Université, INSERM, Centre de Recherches Saint-Antoine, Paris, France; ^2^ Centro de Investigaciones Endocrinológicas “Dr. César Bergadá” (CEDIE), CONICET – FEI – División de Endocrinología, Hospital de Niños Ricardo Gutiérrez, Buenos Aires, Argentina; ^3^ Universidad de Buenos Aires, Facultad de Medicina, Departamento de Biología Celular, Histología, Embriología y Genética, Buenos Aires, Argentina; ^4^ School of BioSciences, The University of Melbourne, Melbourne, VIC, Australia

**Keywords:** activin A, androgen, anti-Müllerian hormone, inhibin B, INSL3, Müllerian ducts, muscle, RXFP2

Hormones produced by the fetal testis are responsible for shaping the male phenotype, as shown by Alfred Jost during the last century (1, 2). Testosterone, produced by fetal Leydig cells, maintains Wolffian ducts and masculinizes the external genitalia; anti-Müllerian hormone (AMH) secreted by Sertoli cells intiates the regression of the Müllerian ducts while another Leydig-cell peptide, Insulin-like peptide 3 (INSL3), induces testicular descent to the scrotum. Receptors for each of these hormones have been identified in target organs. The tools required to study these genes and pathways have been created, providing a solid foundation on which to build new knowledge in this area.

This Research Topic addresses contemporary questions concerning the synthesis and function of fetal testicular hormones. Amato et al. provide an in-depth analysis on the knowledge of the conserved androgen signaling mechanisms as well as the cell- and organ-specific mechanisms illustrated by the androgen-dependent differentiation processes occurring in the Wolffian duct, leading to the formation of the epididymis, vas deferens and seminal vesicle, and in the primordia of the external genitalia. Ipulan-Colet summarizes the role of androgens in driving sexually dimorphic development of the genital tubercle, and uses this framework to scrutinize the different mechanisms potentially involved in the androgen-dependent sexual dimorphism observed in muscle differentiation. The structure, dynamics of expression and regulation of the more recently discovered Leydig cell hormone INSL3 is reviewed by Ivell et al. The authors deal with the mechanisms through which INSL3 and its G-protein coupled receptor, RXFP2, promote testicular descent during fetal life. They also discuss the novel relevance of INSL3 as a biomarker of Leydig cell function *in utero* to its potential to act as a monitor of exposure to environmental endocrine disruptors that may lead to cryptorchidism.

AMH is a dimeric glycoprotein of the transforming growth factor beta (TGFβ) superfamily (3). Members of this family are typically synthesized as large precursors that, after cleavage, produce a small C-terminal active domain and a larger N-terminal domain. In the case of AMH, the N-terminal domain enhances the C-terminal domain activity on the specific type II AMH receptor. Howard et al. review the most recent discoveries on the regulation of the processing of both AMH and its type II receptor. Cate meticulously dissects the signal transduction mechanisms engaged in the AMH-dependent regression of Müllerian ducts in the male fetus, addressing the canonical AMH signaling pathway that involves the specific type II receptor and shared type I receptors leading to the phosphorylation, nuclear translocation and DNA binding of specific Smad proteins. Few effectors are known to mediate AMH action in Müllerian ducts. Based on transcriptome analyses performed in mesenchymal tissue of Müllerian ducts after the start of AMH expression in mouse male fetuses, Mullen et al. identified *Dlx5* and *Dlx6* as potential AMH target genes. Here, they provide experimental evidence that AMH signaling is essential for a sex dimorphic expression of *Dlx5* and *Dlx6* in Müllerian duct mesenchyme, and that disruption of their expression results in persistence of Müllerian derivatives in male mice. Although AMH action takes place very early in fetal development, Sertoli cells continue to produce high amounts of AMH until puberty. Edelsztein et al. review the recent findings related to the molecular mechanisms involved in the strong androgen-mediated downregulation and the weaker estrogen-induced upregulation of testicular AMH expression during puberty. They underscore the importance of understanding these complex regulatory mechanisms for the interpretation of serum AMH as a biomarker in physiological and pathological conditions in boys and adolescents.

A fine-tuned orchestration of regulatory mechanisms is needed for the testis to differentiate and secrete its fetal hormones in an adequate spatiotemporal manner. Viger et al. review the importance of GATA factors for the differentiation of the testis from the gonadal ridge as well as their relevance in the control of fetal Sertoli and Leydig cell gene expression in normal conditions and in human pathology. O’Donnell et al. address the key role of Sertoli cells and activin A for fetal testicular steroidogenesis, arguing that Sertoli cells synthesize testosterone from androstenedione in the fetal mouse testis, and probably also in the human. Lucas-Herald and Mitchell review the role of two Sertoli cell peptides, AMH and inhibin B, during normal male sexual development in the human fetus and their relevance in congenital disorders of sex development.

Given the rise in differences in sexual development and infertility in humans, this timely collection of articles sheds new light of the role of fetal testicular hormones in sexual development and the potential sources of disruption which may be contributing to reduced reproductive health.

## Author contributions

All authors listed have made a substantial, direct and intellectual contribution to the work, and approved it for publication.

## Conflict of interest

The authors declare that the research was conducted in the absence of any commercial or financial relationships that could be construed as a potential conflict of interest.

## Publisher’s note

All claims expressed in this article are solely those of the authors and do not necessarily represent those of their affiliated organizations, or those of the publisher, the editors and the reviewers. Any product that may be evaluated in this article, or claim that may be made by its manufacturer, is not guaranteed or endorsed by the publisher.
